# Whole exome sequencing revealed variants in four genes underlying X-linked intellectual disability in four Iranian families: novel deleterious variants and clinical features with the review of literature

**DOI:** 10.1186/s12920-023-01680-y

**Published:** 2023-10-11

**Authors:** Atefeh Mir, Yongjun Song, Hane Lee, Hossein Khanahmad, Erfan Khorram, Jafar Nasiri, Mohammad Amin Tabatabaiefar

**Affiliations:** 1 Department of Genetics and Molecular Biology, School of Medicine, Isfahan University of Medical Sciences, Isfahan, 81746 73461 Iran; 2 Division of Medical Genetics, 3Billion Inc, Seoul, South Korea; 3 Pediatric Inherited Diseases Research Center, Research Institute for Primordial Prevention of Noncommunicable Disease, Isfahan University of Medical Sciences, Isfahan, Iran; 4 Child Growth and Development Research Center, Research Institute for Primordial Prevention of Non-Communicable Disease, Isfahan University of Medical Sciences, Isfahan, Iran; 5 Deputy of Research and Technology, GenTArget Corp (GTAC), Isfahan University of Medical Sciences, Isfahan, Iran

**Keywords:** XLID, *L1CAM*, *ZDHHC9*, *ATP2B3*, *GLRA2*, *Intellectual disability*, *Exome sequencing*, *Iran*

## Abstract

**Aim and Objective:**

Intellectual disability (ID) is a heterogeneous condition affecting brain development, function, and/or structure. The X-linked mode of inheritance of ID (X-linked intellectual disability; XLID) has a prevalence of 1 out of 600 to 1000 males. In the last decades, exome sequencing technology has revolutionized the process of disease-causing gene discovery in XLIDs. Nevertheless, so many of them still remain with unknown etiology. This study investigated four families with severe XLID to identify deleterious variants for possible diagnostics and prevention aims.

**Methods:**

Nine male patients belonging to four pedigrees were included in this study. The patients were studied genetically for Fragile X syndrome, followed by whole exome sequencing and analysis of intellectual disability-related genes variants. Sanger sequencing, co-segregation analysis, structural modeling, and in silico analysis were done to verify the causative variants. In addition, we collected data from previous studies to compare and situate our work with existing knowledge.

**Results:**

In three of four families, novel deleterious variants have been identified in three different genes, including *ZDHHC9 (*p. Leu189Pro), *ATP2B3 (*p. Asp847Glu), and *GLRA2 (*p. Arg350Cys) and also with new clinical features and in another one family, a reported pathogenic variant in the *L1CAM (*p. Glu309Lys) gene has been identified related to new clinical findings.

**Conclusion:**

The current study's findings expand the existing knowledge of variants of the genes implicated in XLID and broaden the spectrum of phenotypes associated with the related conditions. The data have implications for genetic diagnosis and counseling.

## Introduction

Intellectual disability (ID) is a heterogeneous condition affecting brain development, function, and/or structure. ID has a prevalence of about 2–3% of global populations, and males exceed females by 20–30%, likely due to an enrichment of genes on the X-chromosome that are required for the neurodevelopment and the genetic imbalances in X-chromosomes. The prevalence of X-linked ID (XLID) in males has been estimated at 1 case in 600 to 1000 births. Thus, it is the most frequent cause of ID in males [1, 2]. XLID can be grouped into syndromic and non-syndromic forms. At least 209 different XLID disorders have been described, including 143 syndromic forms [3]. Fragile X syndrome is the most common and most studied XLID syndrome. In the recent decade, remarkable progress has been made in identifying new causative genes and understanding the underlying mechanisms in over 100 XLID conditions [4]. Next-generation sequencing (NGS) is the most powerful technique for identifying new variants and genes in XLID conditions [5]. Despite all advances, more than 80 XLID conditions remain without a molecular diagnosis [6], and identifying disease-causing genes and variants is necessary for precise diagnosis. It would expand the existing knowledge of XLID and the spectrum of phenotypes associated with the identified variants. A vast majority of known causative genes are highly expressed in the brain and involved in different biological functions and pathways [7]. Four of XLID- associated genes involved in neuronal signaling pathways are *L1CAM, ZDHHC9, GLRA2*, and *ATP2B3*. The *L1CAM* gene (MIM No. 308840) encodes a neural cell adhesion molecule involved in cell adhesion dynamics and the generation of transmembrane signals at tyrosine kinase receptors. It is critical in multiple processes during brain development, including neuronal migration, axonal growth and fasciculation, and synaptogenesis [8]. The *ZDHHC9* gene (MIM No. 300646) encodes a palmitoyl-transferase that adds palmitate onto various protein substrates. It is implicated in neurological disorders [9]. The *GLRA2* gene (MIM No. 305990) encodes a glycine receptor responsible for mediating glycine's inhibitory effects in neurons and is widely distributed throughout the CNS, particularly within the hippocampus, spinal cord, and brain stem [10]. The *ATP2B3* gene (MIM No. 300014) encodes an ATP-driven calcium ion pump involved in the maintenance of basal intracellular calcium levels at the presynaptic terminals [11]. Clinical and genetic studies on these genes are few and more studies have led to precise diagnoses of the conditions and the design of new therapeutic approaches; the knowledge could also be helpful in genetic counseling, prenatal diagnosis (PND), pre-implementation genetic diagnosis (PGD), and predict prognosis of the disease.

The present study was launched on four families suspected with XLID. Whole exome sequencing (WES) and clinical evidence were used to identify pathogenic variants in the subjects. Furthermore, precise phenotyping and literature review were performed.

## Material and methods

### Human subject and consent approval

The research was performed according to the Declaration of Helsinki and was approved by the Ethics Committee of the Medical University of Isfahan, Isfahan, Iran (Ethics code: IR.MUI.MED.REC.1400.042). Four families with two or more ID patients suspected with the X-linked mode of inheritance were ascertained from the Isfahan and, Sistan & Balouchestan provinces of Iran. Through genetic counseling, medical history was taken, and pedigrees were drawn by the “Progeny” software (Progeny Software, LLC).

### DNA extraction and molecular testing

Peripheral blood was withdrawn after taking informed written consent from the legal guardians. DNA was extracted using the DNSol Miniprep Kit (provided by ROJETECHNOLOGIES company, Tehran, Iran). All probands were tested for *FMR1* CGG repeats to rule out Fragile X syndrome. It was done using Deviner® Fragile X (*FMR1* Gene) Carrier Screen Kit (provided by KEYSAR Company, Tehran, Iran).

### Library preparation & whole exome sequencing

WES was done by 3Billion Inc. Exome capture was performed using xGen Exome Research Panel v2 (Integrated DNA Technologies, Coralville, Iowa, USA). Sequencing was performed using NovaSeq 6000 (Illumina, San Diego, CA, USA).

### Data analysis

The bases of the sequences were generated and uniquely aligned to the Genome Reference Consortium Human Build 37 (GRCh37) and revised Cambridge Reference Sequence (rCRS) of the mitochondrial genome. The variant interpretation was performed using the EVIDENCE software [12] to prioritize variants and interpreted based on the guideline recommended by the American College of Medical Genetics and Genomics (ACMG) and the Association for Molecular Pathology (AMP) [13] in the context of the patient’s phenotypes. Relevant family history and previous clinical test results were provided through genetic counseling. Only variants deemed to be clinically significant and relevant to the patient's primary clinical indications at the time of variant interpretation were considered.

### Bioinformatics tools

We used the Genome Aggregation Database (gnomAD v2.1.1) for population allele frequency analysis. The potential pathogenicity of the variants was assessed using the following prediction tools: FATHMM & FATHMM-MKL (Functional Analysis through Hidden Markov Models (v2.3), http://fathmm.biocompute.org.uk), LIST-S2 (https://list-s2.msl.ubc.ca/?session=28AB3E5B08FD16AF971162581885ACC2), M-CAP (Mendelian Clinically Applicable Pathogenicity, http://bejerano.stanford.edu/mcap/), Mutation assessor (http://mutationassessor.org/), MutPred (http://mutpred.mutdb.org/), PROVEAN (PROVEAN scores (v1.1)), SIFT (Scale-Invariant Feature Transform, https://sift.bii.a-star.edu.sg) & SIFT4G, MutationTaster (https://www.mutationtaster.org/), BayesDel (addAF and noAF)( https://fenglab.chpc.utah.edu/BayesDel/), MetaLR (e!Ensembl https://useast.ensembl.org/index.html), MetaRNN (http://www.liulab.science/metarnn.html), REVEL (Rare Exome Variant Ensemble Learner, e!Ensembl https://useast.ensembl.org/index.html), DEOGEN2 (http://deogen2.mutaframe.com/).

### Primer designing and sanger validation

All candidate variants were confirmed using Sanger sequencing, and co-segregation analysis was performed on affected and unaffected members of the families. Specific primers for the variants were designed using the Primer3 online tool (Primer3web, version 4.1.0) and validated by online tools such as Primer-BLAST [14], MFEprimer3.1 [15] and SNPCheck (gene tools, SNPCheck V3). The used primers sequences include *L1CAM* (F: CCACGCCCACCATCAAATG, R: CGGTGACATAGTACGCATGC (product size of 177bp)), *ZDHHC9* (F: CTGGGTGGGGAATTGTGTTG, R: GTGCTCATTTCTAACCTGTCCT (product size of 250bp)), *GLRA2* (F: CTCTCTCTCTCAGGTCTCCTATG, R: TCTGAACTGAGGGGCAATCAT (product size of 186 bp)), *ATP2B3* (F: CAACTTCACCAGCATCGTCAA, R: ACCCTCACTCTCACAATCTG (product size of 213 bp)).

### Homology modeling

I-TASSER Web Server (https://zhanggroup.org/I-TASSER/) was used for modeling the 3-dimensional structure of proteins, and the structure refinement was done by Galaxy Refine (https://galaxy.seoklab.org/cgi-bin/submit.cgi?type=REFINE). Also, we used the PROCHECK program to generate the Ramachandran plot for the evaluation of the predicted 3-D structures. PyMOL (Version 2.2.3, Schrödinger, LLC.) software was used for visualization, mutagenesis, and structural analysis. Also, multiple stability prediction tools (SAAFEC-SEQ, mCSM, INPS-3D, and I-Mutant2.0) were used for evaluating the effects of mutations on the proteins’ stabilities.

## Results

### Clinical manifestations

**Fig. 1:**
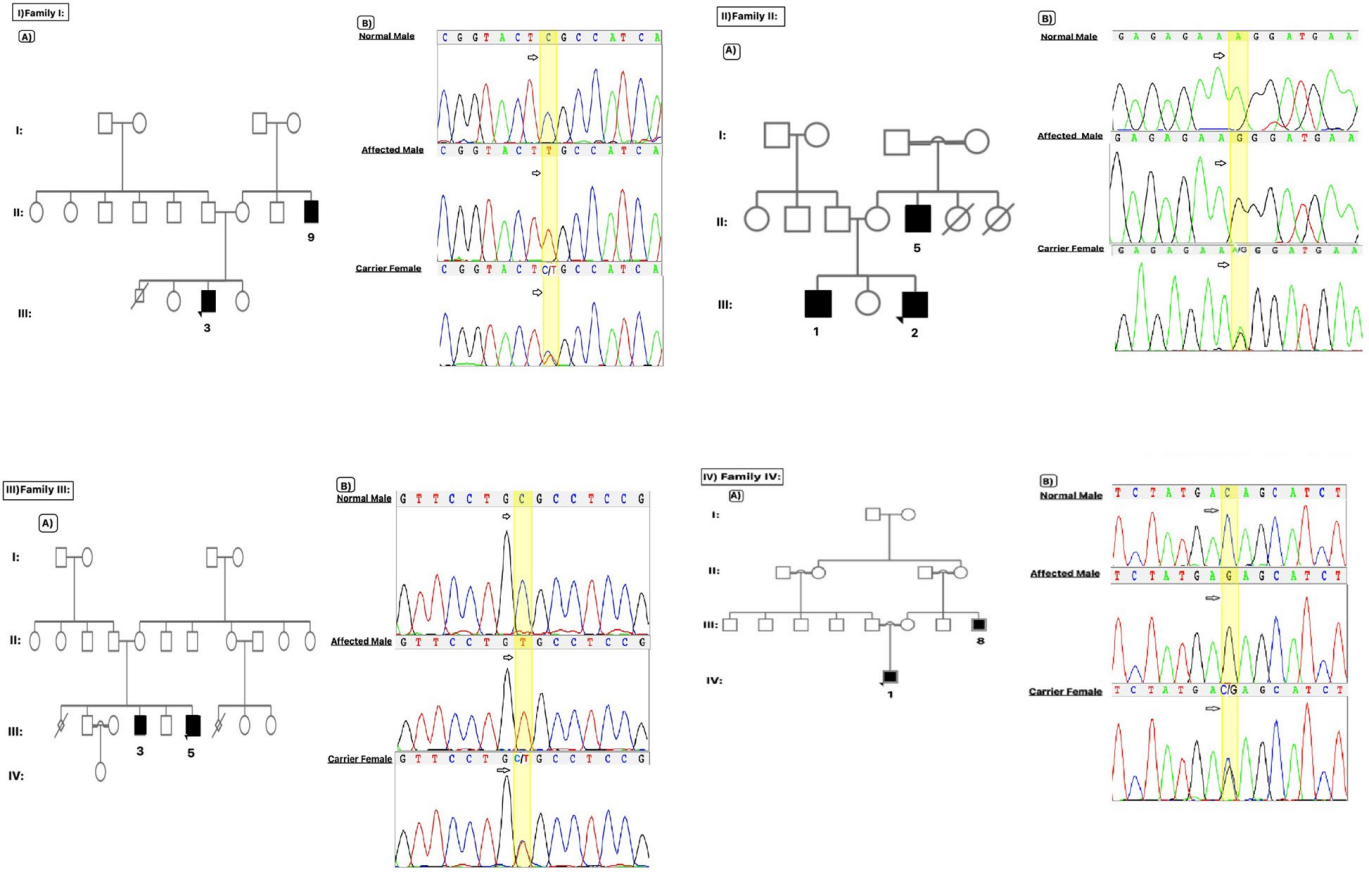
The pedigrees, and chromatogram of studied patients. I: Family I, **A**: pedigree of the family, **B**: Sanger sequencing data of the normal, mutant hemizygous, and heterozygous female carrier of the family; II: Family II, **A**: pedigree of the family, **B**: Sanger sequencing data of the normal, mutant hemizygous and the heterozygous female carrier of the family; III: Family III, **A**: pedigree of the family, **B**: Sanger sequencing data of the normal, mutant hemizygous and the heterozygous female carrier of the family; IV: Family IV, **A**: pedigree of the family, **B**: Sanger sequencing data of the normal, mutant hemizygous and the heterozygous female carrier of the family

Nine severe ID male patients belonging to four families were recruited from the Isfahan and, Sistan & Balouchestan provinces of Iran. Pedigree analysis suggested the possibility of XLID (Fig. 1). The ages of patients ranged from 3 to 40 years at the time of recruitment, and they mainly exhibited severe ID with or without dysmorphic features congenitally. Clinical descriptions of patients are as follows:

- Family I: The proband (Fig. 1-I-A-P(III-3)) was a 14-year-old boy with severe ID who was prenatally diagnosed with hydrocephalus and underwent ventriculoperitoneal shun surgery ten days after birth. He had lower limb muscle weakness in infancy and delayed milestones. He started walking at five years old through occupational therapy. He had no history of difficulties with upper limbs. He suffered from delayed speech and language development and showed poor unclear speech (aphasia). He experienced multiple spasms, such as persistent neck and back muscle spasms that lasted about ten days (spastic paraplegia) and persistent stomach spasms since childhood. He manifested behavior problems, including; aggressive behavior, stressfulness, agitation, and self-injurious behavior (He showed bumps on finger joints due to chewing his fingers). He shows some dysmorphic features such as a long thin face, strabismus (hypertropia type), low-set ears, wide nasal bone, and a severe toe deformity that was corrected by surgery a couple of years ago. The mother had an abortion history of a male fetus in the 5^th^ month of pregnancy for an unknown reason. He has two normal sisters without any medical issues. There are family histories of brain hemangioblastoma and severe ID in the maternal uncle (34 years old).
- Family II: The proband (Fig. 1-II-A-P(III-3)) was a three-year-old boy with severe ID, neurodevelopmental delay, delayed milestones with no speech, and no walking. The Magnetic Resonance Imaging (MRI) showed corpus callosum agenesis and colpocephaly. His parents were not consanguineous. He has an elder brother (8 years old) with a similar condition who suffered from severe ID, developmental delay (delayed milestone; i.e., he started walking at six years old), and speech disorder, and also shows vision problems, persistent leg pains, and muscle weakness beginning from six years old of age. They have a sister with no medical issues. There is a family history of severe ID, speech disorder, and vision problems in the maternal uncle, who is 40 years old and a child of consanguineous marriage.
- Family III: The proband (Fig. 1-III-A-P(III-5)) was a 17-year-old boy with severe ID and aggressive behavior. He had speech and developmental delays diagnosed as pervasive developmental disorders (PDD) by a pediatric neurologist due to behavioral and communication problems. He also shows some facial features such as a broad face, prominent lips, low-set-ears, broad eyebrows, long eyelashes, prominent eyelashes, and a broad nasal tip. His electroencephalogram (EEG) reports were normal. He is not a child of consanguineous marriage. He has an elder brother (29 years old) with similar conditions, including; severe ID and developmental and speech delay. However, there are some differences between these affected brothers in phenotype; the elder brother did not show aggressive behavior, is incommunicative and silent, shows a milder phenotype in facial features, and also experienced epilepsy in childhood, which was controlled by medicine. The mother had a history of abortion in the 3rd month of pregnancy. They had no positive family history of intellectual disability.
- Family IV: The proband (Fig. 1-IV-A-P(IV-1)) was a four-year-old boy with severe ID, neurodevelopmental delay, delayed milestones with no speech, no walking, and hearing impairment. He manifested behavioral problems such as restlessness and crying. He shows dysmorphic facial features such as strabismus, congenital hypotrichosis, and low eyebrow. He had seizures at the age of two that is controlled by medicine. There are some bumps on the skull in touch examinations with no medical diagnosis. EEG showed a severe abnormality, and Auditory Brainstem Response (ABR) showed moderate hearing loss in the right ear and moderate to severe hearing loss in the left ear. Metabolic panel screening was negative. Brain computerized tomography (CT) scan showed coronal and axial thin sections in petrous bone, normal appearance of both external auditory canal, middle ear cavity, ossicular chain, and inner ear structure, no bony erosion, and normal scan of petrous bones. His parents are relatives. He had a family history of severe ID and seizures in the maternal uncle (19 years old), who has a movement disability in the left limbs. He also has facial features such as; a long face, prominent ear, low eyebrow, and broad nasal tip.

**Fig. 2:**
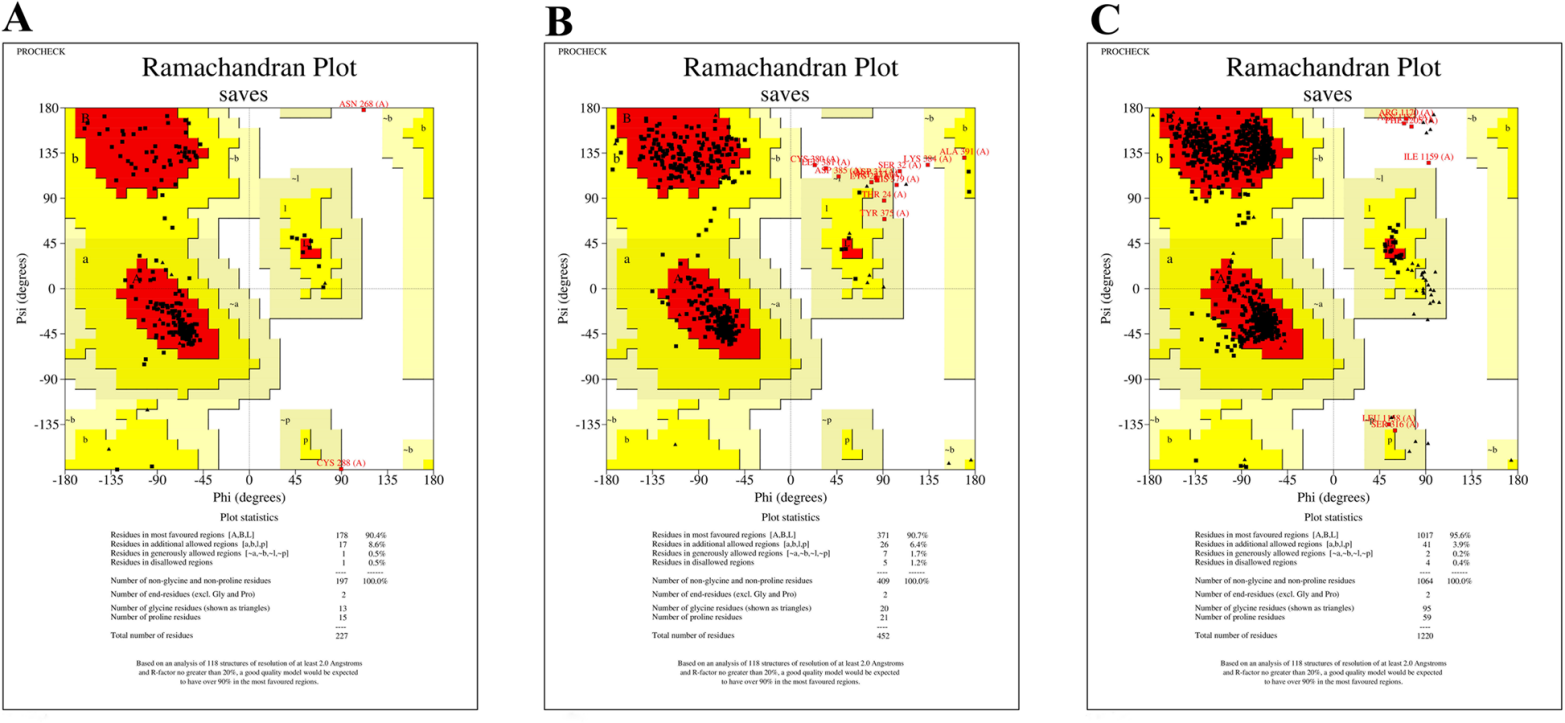
Ramachandran plot of three predicted structures. Plot of ZDHHC9, GLRA2, and ATP2B3 proteins depicted in **A**, **B**, and **C** numbers, respectively. Four colored areas of red, yellow, cream, and white show the most favored region, the additional allowed region, the generously allowed region, and the non-allowed region, respectively

**Fig. 3:**
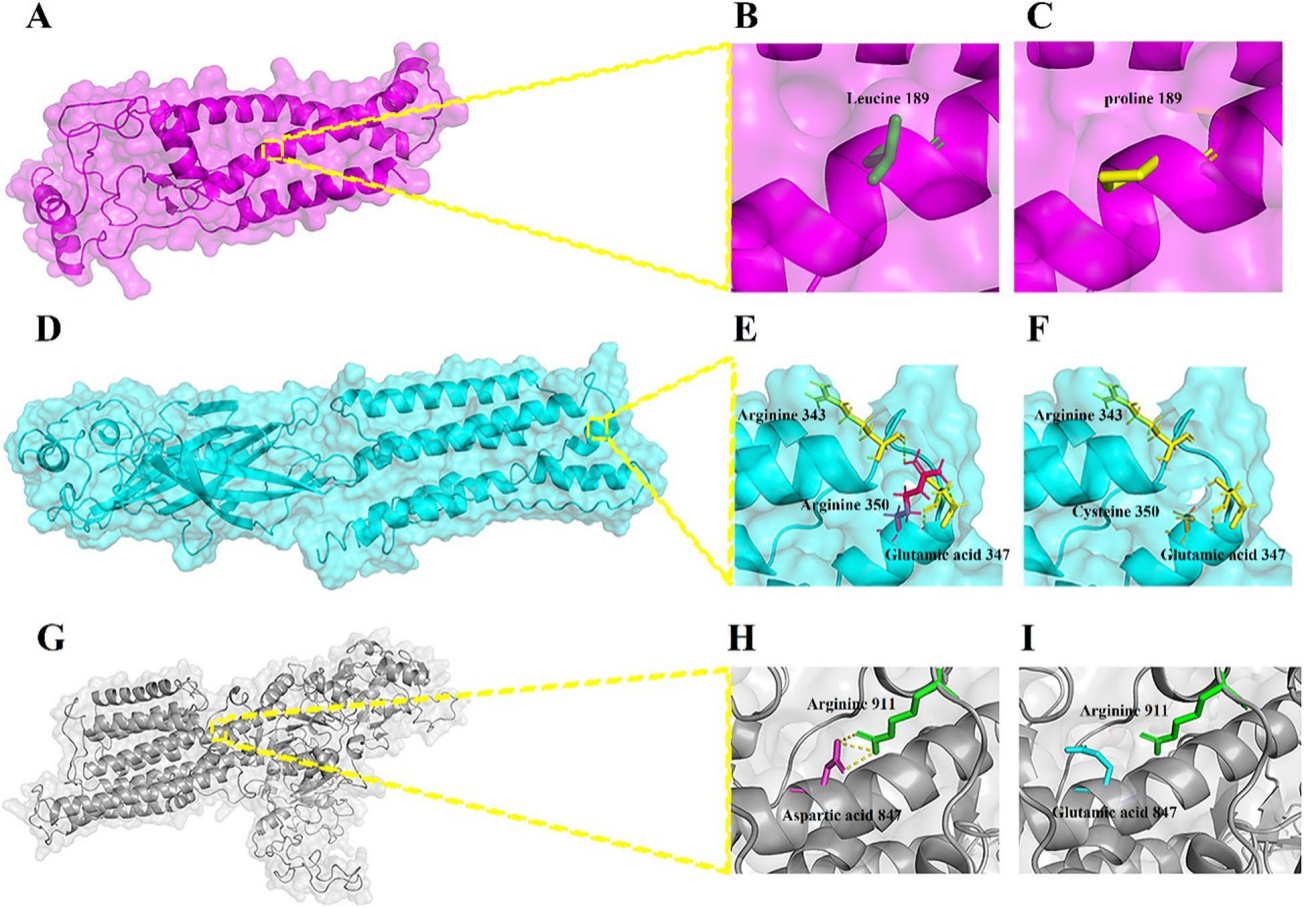
The 3-D structure of the ZDHHC9*,* GLRA2, and ATP2B3 proteins. **A**: The modeled structure of the mutated ZDHHC9 protein containing the p.Leu189Pro variant, the Leucine residue has been substituted with proline 189 (**B**, **C**); due to its irregular geometry, the proline residue destabilizes α-helices and probably disrupts the protein function. **D**: It illustrated the structure of the GLRA2 protein. Figure **E** shows that arginine 350 forms a salt bridge and a hydrogen bond with glutamic acid 347 and a salt bridge with arginine 343 (p.Arg350Cys). As a result of cysteine substitution, all of the mentioned hydrogen bonds are lost (**F**). **G**: The modeled structure of the ATP2B3 protein containing the p.Asp847Glu variant, the aspartic acid residue, has been substituted with glutamic acid 847 (**H**, **I**). As with aspartic acid, glutamic acid is an acidic residue; however, it has a smaller side chain, and due to its distance from the arginine 911 side chain, the salt bridge is broken

### Molecular analysis

Screening for CGG trinucleotide repeats in the 5′ untranslated regions of the *FMR1* gene showed normal ranges of repeat numbers (less than 55). All of the probands were selected for WES, and after WES data analysis, deleterious candidate variants were revealed on the X chromosome; *L1CAM (*ChrX:153,135,577:C > T(GRCh37), NM_001278116.2, c.925G > A, p.Glu309Lys), *ZDHHC9 (*ChrX:128,948,693:A > G(GRCh37), NM_016032.4, c.566T > C, p.Leu189Pro), *GLRA2(*ChrX:14,708,949:C > T(GRCh37), NM_002063.4, c.1048C > T, p.Arg350Cys) and *ATP2B3 (*ChrX:152,823,677:C > G(GRCh37), NM_001001344.2, c.2541C > G, p.Asp847Glu). Sanger sequencing validated candidate variants. In Family I, the variant was found to be co-segregated with the conditions in the proband, and his maternal uncle and mother and one of the proband’s sisters showed carrier status (Fig. 1-I-B). In Family II and IV, the variants were segregated in affected members, while the mothers were heterozygote carriers (Fig. 1-II-B and Fig. 1-IV-B). In Family III, two brothers were hemizygous for the candidate variant, the mother was heterozygote, and the candidate variant was not seen in healthy males in the pedigree (Fig. 1-III-B).

The stability tools predicted all three novel candidate variants to be destabilizing/decreased stability (Table 1). Population frequency databases revealed the NM_016032.4(*ZDHHC9*): c.566T > C and NM_001001344.2(*ATP2B3)*, c.2541C > G were not observed in the gnomAD database. The NM_002063.4(*GLRA2*):c.1048C > T variant was observed at an extremely low frequency in the gnomAD database (total allele frequency: 0.002%).

**Table 1:** The results of prediction servers on protein stability due to three novel deleterious variants

Stability prediction server	INPS-3D	mCSM	SAAFEC-SEQ	I-Mutant2.0
*GLRA2:* (p.Arg350Cys)	Destabilizing	Destabilizing	Destabilizing	Decreased stability
*ZDHHC9*: (p.Leu189Pro)	Destabilizing	Destabilizing	Destabilizing	Decreased stability
*ATP2B3*: (p.Asp847Glu)	Destabilizing	Destabilizing	Destabilizing	Decreased stability

The NM_016032.4(*ZDHHC9*): c.566T > C variant has a damaging effect based on FATHMM-MKL (score = 0.9845), LIST-S2 (score = 0.9765), M-CAP (score = 0.3318), Mutation assessor (score = 4.115), MutPred (score = 0.749), PROVEAN (score = 4.25), SIFT (score = -0.003), SIFT4G (score = -0.031), MutationTaster (score = 1).

Pathogenicity score of the NM_002063.4(*GLRA2*):c.1048C > T variant using in silico prediction tools such as BayesDel addAF (score = 0.2), BayesDel noAF (score = 0.17), MetaLR (score = 0.7), MetaRNN (score = 0.68), REVEL (score = 0.69), DEOGEN2 (score = 0.77), FATHMM (score = 1.68), FATHMM-MKL (score = 0.85), LIST-S2 (score = 0.99), M-CAP(score = 0.38), MutationTaster (score = 1), PROVEAN (score = 4.16, 4.08,4.28), SIFT (score = -0.01, -0.003), SIFT4G (score = -0.005, -0.009) showed this variant as damaging.

The pathogenicity of the NM_001001344.2(*ATP2B3)*, c.2541C > G variant had been analyzed by BayesDel addAF (score = 0.4), BayesDel noAF (score = 0.33), MetaLR (score = 0.93), MetaRNN (score = 0.85), REVEL (score = 0.84), FATHMM (score = 4.49), FATHMM-MKL (score = 0.95), LIST-S2 (score = 0.96), M-CAP (score = 0.45), MutationTaster (score = 0.9999), PROVEAN (score = 3.71) and SIFT (score = -0.001), which suggested it’s the damaging effect.

According to the results of the current study, the NM_016032.4(*ZDHHC9*): c.566T > C and the NM_001001344.2 (*ATP2B3)*, c.2541C > G variants met the criteria of PM1 (located in a mutational hot spot and/or critical and well-established functional domain without benign variation), PM2 (absent from controls or at extremely low frequency if recessive in Exome Sequencing Project, 1000 Genomes or ExAC), PP1 (co-segregation with the disease in multiple affected family members in a gene definitively known to cause the disease) and PP3 (Multiple lines of computational evidence support a deleterious effect on the gene or gene product) and the NM_002063.4(*GLRA2*):c.1048C > T variant had evidence for PM1, PM2, PM5 (novel missense change at an amino acid residue where a different missense change determined to be pathogenic has been seen before), PP1 and PP3 criteria. Therefore, all three novel candidate variants were classified as “likely pathogenic” based on ACMG guideline of variant interpretation [13].

### Homology modeling

The Ramachandran plot of predicted 3-D structures of three proteins is shown in Fig. 2. In general, a good quality model should contain > 90% amino acids in favorable region [16]. As shown in Table 2, more than 90% of the amino acids in all three predicted models are located in the favorable region, so all three can be considered suitable. As a result of the p.Leu189Pro variant in the *ZDHHC9* gene, which was found in Family II, a leucine amino acid (Fig. 3-B) at position 189 of ZDHHC9 protein (Fig. 3-A) has been substituted with a proline (Fig. 3-C). Figure 3-A shows that this residue is located in an alpha helix secondary structure.

**Table 2:** Ramachandran plot statics. As shown, all three predicted structures have > 90% amino acids in the most favored region so they are good structures

Protein	ZDHHC9	GLRA2	ATP2B3
Residue in most favored region	(178) 90.4%	(371) 90.7%	(1017) 95.6%
Residue in additional allowed region	(17) 8.6%	(26) 6.4%	(41) 3.9%
Residue in generously allowed region	(1) 0.5%	(7) 1.7%	(2) 0.2%
Residue in disallowed region	(1) 0.5%	(5) 1.2%	(4) 0.4%

The variant p.Arg350Cys in the GLRA2 protein (Fig. 3-D) in Family III leads to the replacement of arginine 350, which forms salt bridges and hydrogen bonds with glutamic acid 347 and salt bridges with arginine 343 (Fig. 3-E), to the cysteine, and all the mentioned bonds are lost (Fig. 3-F).

Finally, in the ATP2B3 protein (Fig. 3-G), because of the p.Asp847Glu variant in Family IV, an aspartic acid 847 (Fig. 3-H), which is an acidic residue, is replaced with another acidic residue (glutamic acid) (Fig. 3-I). As shown in Fig. (3-H), aspartic acid 847 forms a salt bridge and a hydrogen bond with arginine 911. Due to its smaller side chain, the glutamic acid replacement results in the loss of the abovementioned salt bridge and hydrogen bond (Fig. 3-I). Moreover, several stability tools predicted all of the tree mutations as destabilizing.

## Discussion

X-linked intellectual disability (XLID) is characterized by extensive genetic heterogeneity; to date, variants in more than 100 genes on the X-chromosome are known to be associated with XLID [17]. Here, we investigated four families with XLID conditions using WES and identified novel deleterious variants in three genes with variable clinical findings and a reported deleterious variant with new clinical features.

In Family I, a previously known pathogenic variant (NM_001278116.2: c.925G > A, p. Glu309Lys) in the *L1CAM* gene was identified. The *L1CAM* gene is located near the telomere of the long arm of the X chromosome at Xq28. It consists of 29 exons and encodes the L1 Cell Adhesion Molecule (L1CAM protein), a neuronal cell adhesion molecule that plays an essential role in nervous system development, including neuronal migration and differentiation [8]. To date, 240 different *L1CAM* mutations have been reported, scattered over the entire gene without hot spots, and more than 200 are disease-causing mutations [18]. Mutations in this gene are associated with eight phenotypic conditions and, interestingly, can lead to a clinical spectrum called L1 syndrome that varies not only between families but sometimes also within families. The p.Glu309Lys (c.925G > A) variant in this gene identified in the present study is related to the phenotypic features in two relative patients( Fig. 1-I-A). It is located in exon 8 and affects the Ig-like C2-type 3 ectodomain of the L1CAM protein [8]. The identified p.Glu309Lys (c.925G > A) variant in the *L1CAM* gene was previously described [19, 20], related to MASA (Mental retardation, Aphasia, Shuffling gait, Adducted thumbs) syndrome and is confirming genotype–phenotype correlation in which milder phenotypes are related to the missense mutation in the extracellular domain of the *L1CAM* gene. However, there are phenotypic differences between the previous studies on this deleterious variant and the current study on patients diagnosed with this variant (Table 3). We encountered some new clinical features in the present study, including; behavior problems such as self-injurious, aggressive behavior, agitation, and stressfulness, and some dysmorphic characteristics such as a long thin face, strabismus (hypertropia type), low set ears, wide nasal bone, and severe toe deformity, not previously reported for L1 syndrome or other *L1CAM* gene mutations. These differences can be due to variable expression related to the *L1CAM* gene mutations, which may be caused by modifier genes, epigenetics, or environmental factors. This study confirmed prior knowledge of a broad range of phenotypic manifestations of *L1CAM* gene mutations and the pleiotropic effects of the *L1CAM* gene.

**Table 3:** A review of the literature on the NM_001278116.2(*L1CAM*): c.925G > A (p. Glu309Lys) variant

NM_001278116.2(*L1CAM*): c.925G > A (p. Glu309Lys)		Intellectual disability	Adducted thumbs	Spastic paraplegia	Aphasia	Hydrocephalus	Behavior problems	Dysmorphic features
**Jouet et al. (1995) **[20]		Yes	Yes	Yes	Yes	Yes	-	-
**Straussberg et al. (1991) **[19]		Yes	Yes	Yes	No	-	-	-
**Patients in this study**	**A**	Yes	No	Yes	Yes	Yes	Yes	Yes
	**B**	Yes	No	Yes	Yes	Yes	Yes	Yes

In the second family (Family II), a novel deleterious hemizygous missense variant in the *ZDHHC9* gene (NM_016032.4: c.566T > C, p.Leu189Pro) in two brothers with severe ID, developmental delay, and speech disorder (Fig. 1-II-A) was identified. The *ZDHHC9* gene is located on chromosome Xq26.1 and consists of 12 exons and encoded five transcripts. It encodes an integral membrane protein member of the zinc finger DHHC domain-containing protein family which forms a complex with golgin subfamily A member seven and functions as a palmitoyl transferase [9]. The ZDHHC enzymes are implicated in several neurological and neurodevelopmental disorders. Loss-of-function mutations in the *ZDHHC9* gene have been identified in XLID patients and are related to increased seizure risk [21]. According to the Human Gene Mutation Database (HGMD), LOVD^3^, ClinVar, and literature review, to date, about 18 pathogenic/likely pathogenic mutations have been identified, all of which are related to intellectual disability except one is related to an abnormality of the nervous system (Table 4). Hence, the *ZDHHC9* gene is frequently associated with a syndromic condition called “Raymond-type X-linked syndromic intellectual developmental disorder (MRXSR, OMIM: 300,799)” that is characterized by mild to severe intellectual disability, speech and language difficulties, and additional variable features including marfanoid habitus, epilepsy, facial dysmorphism, hypotonia, and behavioral problems. In family II, the c.566T > C (p. Leu189Pro) variant of the *ZDHHC9* gene was identified, which is located on exon seven and in the DHHC domain that is required for palmitoyl transferase activity [9]. As a result of this study, this mutation causes the enzyme aberrant activity, leading to the disease conditions. In this Family, two brothers and their maternal uncle were studied (Fig. 1-II-A), all three patients suffer from severe ID, developmental delay, and speech disorder that met the primary phenotype of the MRXSR, but there are some additional and variable clinical manifestations in these patients. The proband shows agenesis of the corpus callosum and Colpocephaly which is a new and previously unreported symptom in this condition. His elder brother has new clinical features, including vision problems, persistent leg pains, and muscle weakness beginning at six years of age. A similar vision problem has been encountered in their maternal uncle. These differences in phenotype between previous studies and within the presented family signify variable expression and pleiotropic effects of the *ZDHHC9* gene (Table 4). Notably, vision and muscular problems may be due to the progression of the disorder by age (age-related onset). As few clinical features have been reported related to the variants of this gene, the genotype–phenotype correlations cannot be a result at this stage.

**Table 4:** A review of the literature and databases on the known variants of the *ZDHHC9* gene

Variant		Type of Mutation	Protein Change	ACMG Classification	Related Condition	ID^*a*^	SD^*a*^	A^*a*^	S^*a*^	H^*a*^	DF^*a*^	BP^*a*^	Other Clinical Findings	References
**NM_016032.3(ZDHHC9): c.145T > C**		Missense	p.Cys49Arg	Pathogenic	Not Provided	N/R^*^	N/R	N/R	N/R	N/R	N/R	N/R	-	Hu (2016) Mol Psychiatry **21,** 133 [22]
**NM_016032.4(ZDHHC9):c.251T > C**		Missense	p.Leu84Ser	Likely Pathogenic	Not Provided	N/R	N/R	N/R	N/R	N/R	N/R	N/R	-	ClinVar (RCV000484510.1)
**NM_016032.4(ZDHHC9):c.268G > A**		Missense	p.Asp90Asn	Likely Pathogenic	Syndromic X-Linked Intellectual Disability Raymond Type	N/R	N/R	N/R	N/R	N/R	N/R	N/R	-	ClinVar (RCV000760177.1)
**NM_016032.4(ZDHHC9):c.286C > T**		Missense	p.Arg96Trp	Pathogenic/Likely Pathogenic	Syndromic X-Linked Intellectual Disability Raymond Type	Yes	N/R	N/R	Yes	N/R	N/R	N/R	Global Developmental Delays without Marfanoid habitus, Structural Brain Abnormalities	Tzschach (2015) Eur J Hum Genet **23 **[17]
**NM_016032.4(ZDHHC9):c.442C > T**	**Patient 1**	Missense	p.Arg148Trp	Pathogenic/Likely Pathogenic	Syndromic X-Linked Intellectual Disability Raymond Type, Intellectual Disability	Yes	N/R	Yes	N/R	N/R	Yes	N/R	Developmental Delay, Mild Fixed Flexion Deformity at the Elbows, Large Ears, Long Fingers and Toes, Pes Planus	Raymond (2007) Am J Hum Genet **80,** 982 [23]
	**Patient 2**												Developmental Delay, Large Ears, Long Fingers and Toes, Pes Planus	
**NM_016032.4(ZDHHC9):c.448C > T**		Missense	p.Pro150Ser	Pathogenic	Syndromic X-Linked Intellectual Disability Raymond Type	Yes	N/R	Yes	N/R	N/R	N/R	N/R	Joint Hypermobility, Pectus Excavatum, Long Digits, Delayed Sitting, Adducted Thumbs at Birth that resolved spontaneously, Long Face, Strabismus, Prominent Ears, Long, Thin Limbs, 5th-Finger Camptodactyly, Long Toes with Camptodactyly	Raymond (2007) Am J Hum Genet **80,** 982 [23]
**NM_016032.4(ZDHHC9):c.496G > A**		Missense	p.Asp166Asn	Likely Pathogenic	Syndromic X-Linked Intellectual Disability Raymond Type	N/R	N/R	N/R	N/R	N/R	N/R	N/R	-	ClinVar (RCV001754546.1)
**NM_016032.3( ZDHHC9): c.892C > T**		Missense	p.Arg298Gln	Pathogenic	Moderate Intellectual Disability with Speech Delay	Yes	Yes	N/R	Yes	N/R	N/R	N/R	MRI Brain: Retro-Cerebellar CSF Fluid Collection	Bowling (2017) Genome Med **9,** 43 [24]
**NM_016032.3( ZDHHC9): c.892C > T**		Nonsense	p.Arg298*	Pathogenic	Intellectual Disability	Yes	N/R	N/R	N/R	N/R	N/R	Yes	Limited Extension of the Elbows and Metacarpophalangeal Joints, Acrocyanosis, Dysplastic Corpus Callosum, Lingual Fasciculation	Masurel-Paulet (2014) Am J Med Genet A **164,** 789 [25]
**NM_016032.4(ZDHHC9):c.167 + 5G > C**	**Patient 1**	Splicing	p.Thr11Profs833	Pathogenic	Not Provided	Yes	Yes	Yes	N/R	N/R	Yes	Yes	Developmental Delay, Pectus Carinatum, Pes Planus, Thin Facial Features	Raymond (2007) Am J Hum Genet **80,** 982 [23]
	**Patient 2**													
**NM_016032.3 ( ZDHHC9):c.172-175dup**	**1 (Two sibs)**	Splicing	p.Tyr59Serfs*33	Pathogenic	Not Provided	Yes	Yes	N/R	N/R	Yes	N/R	N/R	Developmental Delay and Moderate Learning Disability, a High Forehead, Severe Constipation, Hypertelorism	Raymond (2007) Am J Hum Genet **80,** 982 [23]
	**2 (Maternal uncle)**												No Walking, Need Full-Time Care	
**NM_016032.3( ZDHHC9):c.361C > T**		Missense	p.(Arg121Ter)	Likely Pathogenic	Not Provided	N/R	N/R	N/R	N/R	N/R	N/R	N/R	-	LovD3 (https://databases.lovd.nl/shared/variants/ZDHHC9/unique)
**NM_016032.3( ZDHHC9): c.878_879insA**		Frameshift (Small Insertion)	p.Ser294Glnfs*26	Likely Pathogenic	Not Provided	Yes	N/R	N/R	N/R	N/R	N/R	N/R	-	Grozeva (2015) Hum Mutat **36,** 1197 [26]
**NM_016032.4(ZDHHC9):c.777 + 1G > A**		Splicing	-	Likely Pathogenic	Abnormality of the Nervous System	N/R	N/R	N/R	N/R	N/R	N/R	N/R	-	Retterer (2016) Genet Med **18,** 696
**NM_016032.3 (ZDHHC9):c.487 + 5_487 + 19del**		Small Deletion	-	Likely Pathogenic	Not Provided	Yes	N/R	N/R	N/R	N/R	N/R	N/R	Global Developmental Delay, Gait Ataxia, Macrotia, High Narrow Palate, Hypertonia, Hyperreflexia	Anazi (2017) Hum Genet **136,** 1419 [27]
**6-31kb DEL, EX10-11DEL**		Gross Deletion	-	Pathogenic	Mental Retardation, X-linked	Yes	N/R	N/R	N/R	N/R	N/R	N/R	-	Boone (2010) Hum Mutat **31,** 1326 [28]
**2-KB DEL, EX6-7DEL**		Gross Deletion	-	Pathogenic	Intellectual Disability	Yes		No	N/R	N/R	Yes	N/R	Elongated and Down‐Slanting Palpebral Fissures and High Hairline	Schirwani (2018) Am J Med Genet A **176,** 1238 [29]
**Insertion of 4.68Mb**		Gross Insertion	-	Likely Pathogenic	Not Provided	Yes	N/R	N/R	N/R	N/R	N/R	N/R	Short Stature, Microcephaly	Willemsen (2012) Eur J Med Genet **55,** 586 [30]
**NM_016032.4 (ZDHHC9): c.566T > C**	**1 (proband)**	Missense	p.Leu189Pro	Likely Pathogenic	Syndromic X-Linked Intellectual Disability Raymond Type	Yes	Yes	No	No	No	No	No	Developmental Delay, Vision Problem (in older brother and maternal uncle), Muscular Weakness (in older brother), Brain Tumor (in proband)	Current Study
	**2 (older brother)**													
	**3 (maternal uncle)**													

^a^*N/R* Not reported, *ID* Intellectual disability, *SD* Speech disorder, *A* Arachnodactyly, *S* Seizures, *H* Hypotonia, *DF* Dysmorphic features, *BP* Behavioral problem

In the third family (Family III), a novel deleterious hemizygous missense variant in the *GLRA2* gene (NM_002063.4:c.1048C > T, p.Arg350Cys) was identified. The *GLRA2* gene is located on Xp22.2, consists of 13 exons, and encodes eight transcripts. *GLRA2* is a protein-coding gene that encodes the alpha subunit of the glycine receptor; which are widely distributed throughout the CNS, particularly within the hippocampus, spinal cord, and brain stem. It plays a role in the down-regulation of neuronal excitability and contributes to generating inhibitory postsynaptic currents [10]. Recent investigations have noted that missense variants in this gene can result in a loss, gain, or altered function of the encoded protein. In turn, missense variants are likely to either negatively or positively deregulate cortical progenitor homeostasis and neuronal migration in the developing brain, leading to changes in cognition, learning, and memory [31]. The most associated disorder with *GLRA2* is X-Linked Intellectual Developmental Disorder, Syndromic, Pilorge Type (MRXSP, OMIM: 301,076). The MRXSP is characterized by a global developmental delay with variably impaired intellectual development, speech delay, behavioral abnormalities, autism spectrum disorder (ASD), and more variable features, including motor incoordination, seizures, and ocular abnormalities. Based on our knowledge, to date, about 12 pathogenic/likely pathogenic variants have been reported according to literature, HGMD, LOVD^3^, and ClinVar (Table 5), most of which are related to MRXSP or ASD. Here, we identified a novel deleterious hemizygous missense variant (NM_002063.4:c.1048C > T, p.Arg350Cys) in this gene in a family with two affected boys with severe ID. They suffered developmental and speech delays, but some differences were found, including seizures in the elder brother and behavior problems in the younger one. In addition, Piton et al. (2011) previously described a pathogenic mutation in a similar amino acid codon (NM_002063.4(*GLRA*2):c.1049G > T, p.Arg350Leu), related to ASD [32]. After a while, Zhang et al. (2017) showed that the R350L mutation altered glycine receptor channel properties and kinetics, with increased inhibitory postsynaptic current (IPSC) and decreased decay times. The reduced decay times lead to longer duration of active periods, and increased conductance of the mutant channel indicated that the R350L mutation results in a gain-of-function effect [33]. In line with Piton et al. (2011) study, the two brothers of the current research in Family III also showed PDD, a subtype or milder type of ASD. The differences in phenotypic manifestation within these two brothers and between other studies may be due to the gene's variable expression or modifier genes and pleiotropic effects. We also encountered some facial features that have been reported for a likely pathogenic variant of this gene in ClinVar submission (ClinVar: RCV001813921.1), including some minor features: a broad face, prominent lips, broad eyebrows, long eyelashes, prominent eyelashes, and a broad nasal tip, which can expand the spectrum of clinical manifestations associated with the disorder. On the other hand, there are few detailed clinical features that have been reported related to this gene's variants, and the lack of such clinical details makes it impossible to find the genotype–phenotype correlations regarding this gene.

**Table 5:** A review of literature and databases on the variants of the *GLRA2* gene

Variant		Type of Mutation	Protein Change	ACMG Classification	Related Condition	ID^*a*^	GDD^*a*^	SD^*a*^	BP^*a*^	Other Clinical Findings	Reference
**NM_002063.4(GLRA2):c.16C > G**		Missense	p. val6leu	Pathogenic	Autism Spectrum Disorder	N/R^a^	N/R	N/R	Yes	Autism Spectrum Disorder	Iossifov (2014) Nature 515, 216 [34]
**NM_002063.4(GLRA2):c.140T > C**		Missense	p.Phe47Ser	Likely Pathogenic​	Not Provided	N/R	N/R	N/R		Global Developmental Delay, Seizure, Infantile Spasms, Nystagmus, Strabismus, Hyperactivity, Sleep Disturbance, Normal Interictal EEG	ClinVar (RCV001810525.1)
**NM_002063.4(GLRA2):c.407A > G**		Missense	p.Asn136Ser	Pathogenic	Intellectual Developmental Disorder, X-Linked, Syndromic, Pilorge Type	N/R	N/R	N/R	Yes	Autism Spectrum Disorder	Pilorge (2016) Mol Psychiatry 21, 936 [35]
**NM_002063.4(GLRA2):c.458G > A**		Missense	p.Arg153Gln	Pathogenic	Intellectual Developmental Disorder, X-Linked, Syndromic, Pilorge Type	N/R	N/R	N/R	Yes	Autism Spectrum Disorder	Pilorge (2016) Mol Psychiatry 21, 936[35]
**NM_002063.4(GLRA2):c.718A > G**		Missense	p.Lys240Glu	Likely Pathogenic	Autism Spectrum Disorder and Developmental Disorder	N/R	N/R	N/R	N/R	-	Chen X (2022) Front Mol Neurosci 15 [36]
**NM_002063.4(GLRA2):c.777C > G**		Missense	p.Ile259Met	Likely Pathogenic	Autism Spectrum Disorder	N/R	N/R	N/R	N/R	-	Marcogliese (2022), Cell Report 15;38 [37]
**NM_002063.4(GLRA2):c.754C > T**		Missense	p.Arg252Cys	Likely Pathogenic	Autism Spectrum Disorder	N/R	N/R	N/R	N/R	-	Marcogliese (2022), Cell Report 15;38 [37]
**NM_002063.4(GLRA2):c.1049G > T**		Missense	p.Arg350Leu	Pathogenic	Intellectual Developmental Disorder, X-Linked, Syndromic, Pilorge Type	N/R	N/R	N/R	Yes	Autism Spectrum Disorder	Piton A (2011) Mol Psychiatry 16 (8) [32]
**NM_002063.4(GLRA2):c.862G > A**		Missense	p.Ala288Thr	Likely Pathogenic	Not Provided	N/R	N/R	N/R	N/R	Seizure, Myoclonus, Cerebellar Ataxia, Developmental Regression	ClinVar (RCV001813918.1)
**NM_002063.4(GLRA2):c.1199C > T**		Missense	p.Pro400Leu	Likely Pathogenic	Not Provided	N/R	N/R	Yes	Yes	Seizure, Disorder of Language, Febrile Seizure (within the age range of 3 months to 6 years), Developmental Regression, Delayed Speech and Language Development, Anxiety, Obesity, Broad Face, Widow's Peak, Broad Eyebrow, Long Eyelashes, Prominent Eyelashes, Broad Nasal Tip, EEG Abnormality, Alpha-EEG	ClinVar(RCV001813921.1)
**NM_002063.4(GLRA2):c.887C > T**		Missense	p.Thr296Met	Likely Pathogenic	Autism Spectrum Disorder	N/R	N/R	N/R	N/R	-	Marcogliese (2022), cell Report 15;38 [37]
**NM_002063.4(GLRA2):c.1334G > A**		Missense	p.Arg445Gln	Likely Pathogenic	Not Provided	Yes	Yes	Yes	Yes	Seizure, Autistic Disorder, Cognitive Impairment, Short Attention Span, Sleep Disturbance, Downslanted Palpebral Fissures	ClinVar (RCV001813920.1)
**NM_002063.4(GLRA2): c.1048C > T**	**Patient 1**	Missense	p.Arg350Cys	Likely Pathogenic	Intellectual Developmental Disorder, X-Linked, Syndromic, Pilorge Type	Yes	Yes	Yes	Yes	Seizures, PDD, Some Facial Features such as; a Broad Face, Prominent Lips, Broad Eyebrows, Long Eyelashes, Prominent Eyelashes, and a Broad Nasal Tip. Normal EEG (in proband)	Current Study
	**Patient 2**										

^a^*N/R* Not reported, *ID* Intellectual disability, *GDD* Global developmental delay, *SD* Speech disorder, *BP* Behavioral problem

Family IV represents a novel deleterious hemizygous missense variant in the *ATP2B3* gene. The *ATP2B3* gene is located on Xq28 and consists of 26 exons. The *ATP2B3* gene encodes ATPase Plasma Membrane Ca2 + , Transporting three involved in the maintenance of basal intracellular Calcium levels at the presynaptic terminals [38]. The most condition related to the *ATP2B3* gene is X-linked Cerebellar ataxia-1 (SCAX1, OMIM: 302,500), an X-linked recessive neurologic disorder characterized by hypotonia at birth, delayed motor development, gait ataxia, difficulty standing, dysarthria, and slow eye movements. Brain MRI shows cerebellar ataxia. To our knowledge, 10 pathogenic/likely pathogenic mutations in this gene have been reported according to literature, HGMD, LOVD^3^, and ClinVar databases (Table 6) related to SCAX1, ataxic disorders, and other neurological disorders. The studied family (Family IV) resulted in the NM_001001344.2, c.2541C > G, p.Asp847Glu variant on the *ATP2B3* gene. Clinical findings matched other studies on *ATP2B3* gene variants related to SCAX1 (Table 6). The proband and his maternal uncle (Fig. 1-IV-A) both suffer from severe ID and seizure. The different clinical manifestations in the proband include neurodevelopmental delay, delayed milestones, no speech, no walking, hearing impairment, agitation, and excessive crying. He shows dysmorphic facial features such as strabismus, congenital hypotrichosis, and low eyebrow. His maternal uncles have movement disability in the left limb that began after multiple seizures early after birth. He also shows facial features such as a long face, prominent ear, low eyebrow, and broad nasal tip. Additionally, as shown in Table 6, missense changes are a common disease-causing mechanism in this gene. Another key finding in this study was the existence of subtle differences in some clinical features that suggest variable expression between and within families with the same gene defects. Again, few clinical features have been reported related to the variants of this gene, and the genotype–phenotype correlations cannot be resolved at the moment.

**Table 6:** A review of literature and databases on the variants of the *ATP2B3* gene

Variant		Type of Mutation	Protein Change	ACMG Classification	Related Condition	ID^*a*^	GDD^*a*^	SD^*a*^	BP^*a*^	Other Clinical Findings	Reference
**NM_001001344.3(ATP2B3):c.130G > A**		Missense	p.Glu44Lys	Likely Pathogenic	Spastic Ataxia	N/R^a^	N/R	N/R	N/R	N/R	ClinVar (RCV001647254.1)
**NM_001001344.3(ATP2B3):c.197C > T**		Missense	p.Ser66Leu	Likely Pathogenic	Arthrogryposis Multiplex Congenita, Fetal Akinesia Deformation Sequence 1	N/R	N/R	N/R	N/R	N/R	ClinVar(RCV000855493.1), Pergande(2020) Genet Med 22(3) -511 [39]
**NM_001001344.3(ATP2B3):c.1445G > A**		Missense	p.Arg482His	Pathogenic	Cerebellar ataxia, X-linked	N/R	Yes	N/R	N/R	Generalized Hypotonia, Cerebellar Ataxia	Calì (2015) J Biol Chem 290, 16,132 [38]
**NM_001001344.3(ATP2B3): c.1610G > A**		Missense	p.Arg537His	Pathogenic	Abnormality of the Nervous System	N/R	N/R	N/R	N/R	N/R	Retterer (2016) Genet Med 18, 696 [40]
**NM_001001344.3(ATP2B3): c.1678C > G**		Missense	p.Pro560Ala	Pathogenic	Autism Spectrum Disorder	N/R	N/R	N/R	N/R	N/R	Al-Mubarak (2017) Sci Rep 7, 5679 [41]
**NM_001001344.3(ATP2B3):c.2197G > A**		Missense	p.Gly733Arg	Pathogenic	Cerebellar Ataxia	Yes	Yes	Yes	N/R	Psychomotor Retardation, Generalized Hypotonia, Hyporeflexia, Dysmetria and Trunk Ataxia, Exotropia and Nystagmus, Skin & Joint Hyperlaxity, Mild Dorsal Kyphosis, Structural Brain Anomalies	Vicario (2017) Biochim Biophys Acta 1863, 3303 [42]
**NM_001001344.3(ATP2B3):c.2770A > G**		Missense	p.Thr924Ala	Likely Pathogenic	X-linked Progressive Cerebellar Ataxia	Yes	Yes	Yes	N/R	Cognitive Impairment, Psychomotor Retardation, Inability to Walk, Limb Tremor	ClinVar (RCV001420157.1)
**NM_001001344.3(ATP2B3):c.3320G > A**		Missense	p.Gly1107Asp	Pathogenic /Likely Pathogenic	X-linked Progressive Cerebellar Ataxia,	N/R	N/R	N/R	N/R	N/R	Zanni (2012) Proc Natl Acad Sci U S A 109, 14,514 [43]
**NM_001001344.3(ATP2B3):c.3338C > T**		Missense	p.Thr1113Met	Likely Pathogenic	Epileptic Encephalopathy with Infantile Spasms	N/R	N/R	N/R	N/R	N/R	Helbig (2016) Genet Med 18, 898 [44]
**NM_001001344.3(ATP2B3):c.3594G > T**		Missense	p.Lys1198Asn	Pathogenic /Likely Pathogenic	X-linked progressive cerebellar ataxia	Yes	Yes	N/R	N/R	Microcephaly, Abnormal Cerebral Cortex Morphology, Hypotonia, Muscular Atrophy, Carious Teeth, Oral-Pharyngeal Dysphagia	Charng (2016) BMC Med Genomics 9, 42 [45]
**NM_001001344.2(ATP2B3), c.2541C > G**	**Patient A**	Missense	p.Asp847Glu	Likely Pathogenic	X-linked Progressive Cerebellar Ataxia	Yes	Yes	Yes	Yes	Delayed in Milestones, no Walking, Hearing Impairment, Agitation and Crying, Dysmorphic Facial Features such as Strabismus, Congenital Hypotrichosis, and Low Eyebrow, Seizure, Severe Abnormality in EEG	Current Study
	**Patient B**										

^a^*N/R* Not reported, *ID* Intellectual disability, *GDD* Global developmental delay, *SD* Speech disorder, *BP* Behavioral problem

## Conclusion

In conclusion, this study revealed three novel deleterious variants in three known genes on X- chromosomes by whole exome sequencing and described novel clinical findings in four unrelated families with XLID disorders. The results broaden the mutational and clinical spectrum of four rare XLID conditions and provide insights into this highly heterogeneous disorder. New mutational reports of families with detailed clinical descriptions will add to the existing knowledge and help to a comprehensive and clear picture of the genetic landscape of XLID.

## Data Availability

All data generated or analyzed during this study are included in this published article and the raw data that support the findings of this study are available from the corresponding author upon reasonable request. In addition, the datasets generated and/or analyzed during the current study are available in the ClinVar repository with accession numbers as: VCV000838485.11, VCV001705288.1, VCV001705404.3, VCV001705366.1.
