# Correction: whole exome sequencing revealed variants in four genes underlying X-linked intellectual disability in four Iranian families: novel deleterious variants and clinical features with the review of literature

**DOI:** 10.1186/s12920-025-02100-z

**Published:** 2025-02-27

**Authors:** Atefeh Mir, Yongjun Song, Hane Lee, Hossein Khanahmad, Erfan Khorram, Jafar Nasiri, Mohammad Amin Tabatabaiefar

**Affiliations:** 1 Department of Genetics and Molecular Biology, School of Medicine, Isfahan University of Medical Sciences, Isfahan, 81746 73461 Iran; 2 Division of Medical Genetics, 3Billion Inc, Seoul, South Korea; 3 Pediatric Inherited Diseases Research Center, Research Institute for Primordial Prevention of Noncommunicable Disease, Isfahan University of Medical Sciences, Isfahan, Iran; 4 Child Growth and Development Research Center, Research Institute for Primordial Prevention of Non- Communicable Disease, Isfahan University of Medical Sciences, Isfahan, Iran; 5 Deputy of Research and Technology, GenTArget Corp (GTAC), Isfahan University of Medical Sciences, Isfahan, Iran

**Correction to: Mir et al. BMC Medical Genomics (2023) 16:239**

10.1186/s12920-023-01680-y.

Following the publication of the Original Article, a reader identified an error concerning Fig. 1. The error occurred due to an unintentional mistake during the image collage-making; the family “I” chromatogram was inadvertently duplicated for family “III”. The authors acknowledged the error and affirmed that the errors did not impact the calculations or the interpretation of the article’s results.

The incorrect figure is as follows:


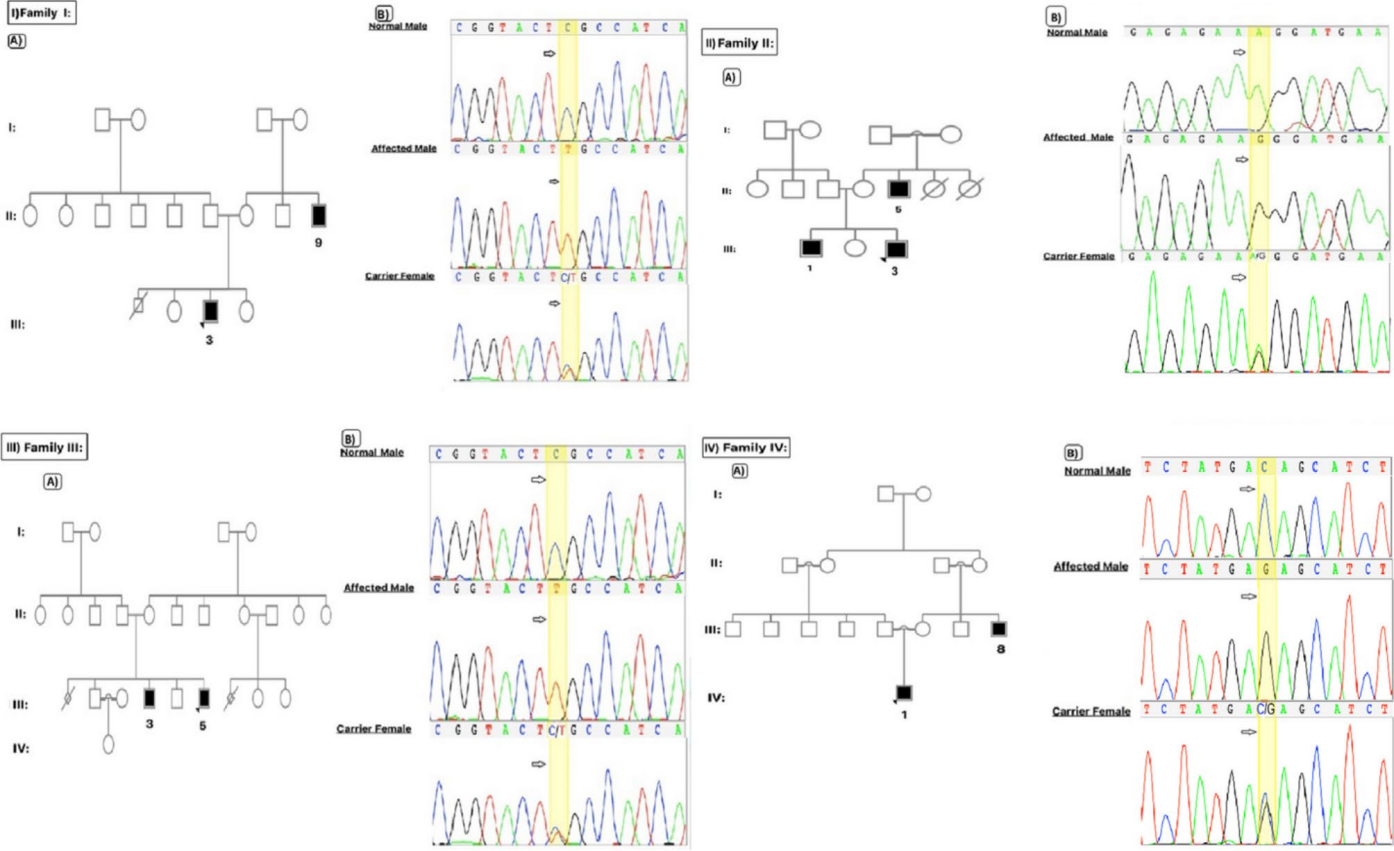


The correct figure is as follows:


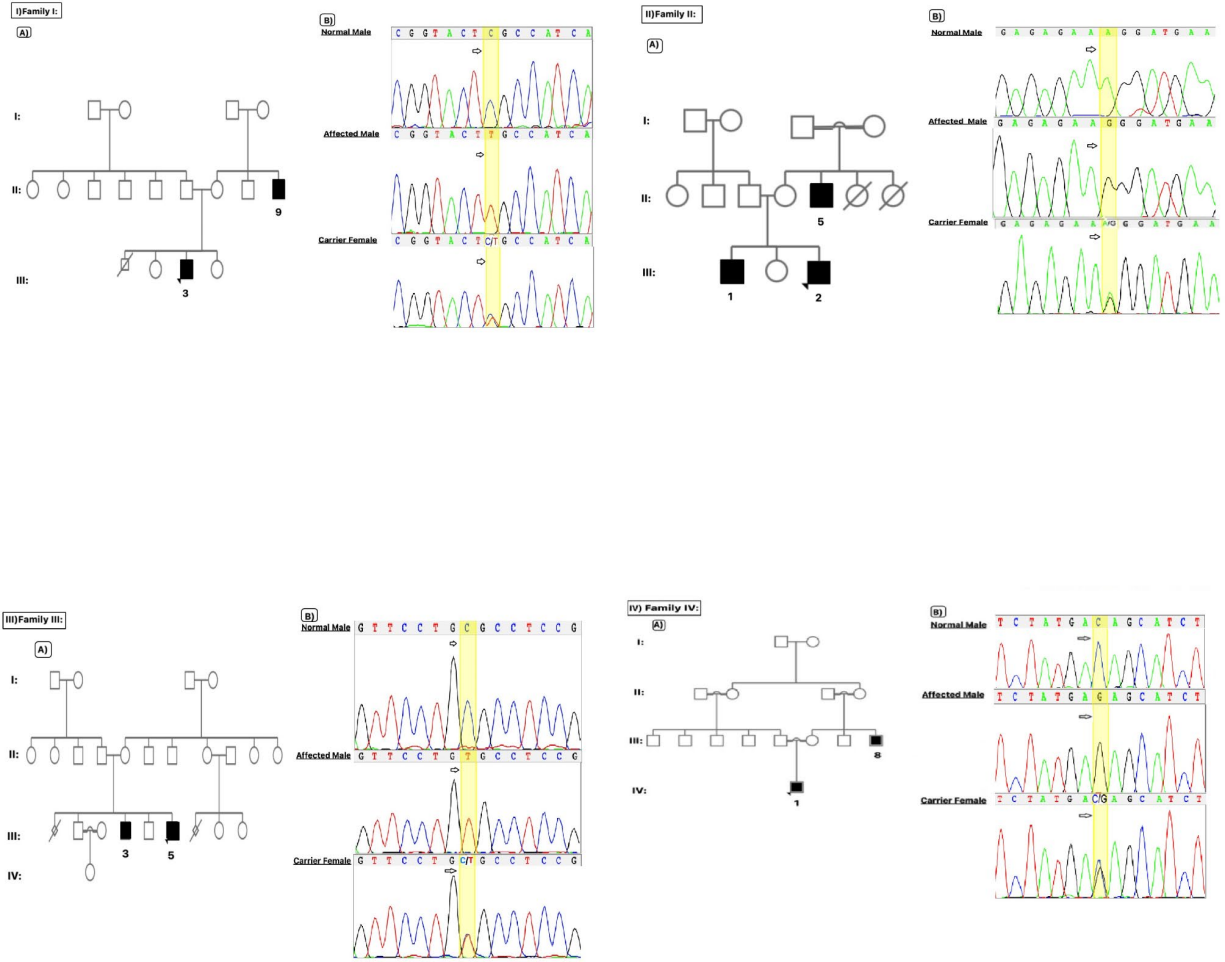


The authors thank the reader for pointing out the error.

The Original Article has been corrected.

